# Identification of Cancer Cell-Line Origins Using Fluorescence Image-Based Phenomic Screening

**DOI:** 10.1371/journal.pone.0032096

**Published:** 2012-02-23

**Authors:** Jun-Seok Lee, Yun Kyung Kim, Han Jo Kim, Siti Hajar, Yee Ling Tan, Nam-Young Kang, Shin Hui Ng, Chang No Yoon, Young-Tae Chang

**Affiliations:** 1 Biomolecules Function Research Center, Korea Institute of Science and Technology, Seoul, South Korea; 2 Center for Neuro-Medicine, Brain Science Institute, Korea Institute of Science and Technology, Seoul, South Korea; 3 Department of Chemistry & Med Chem Program, Life Sciences Institute, National University of Singapore, Singapore, Singapore; 4 Lab of Bioimaging Probe Development, Singapore Bioimaging Consortium, Agency for Science, Technology and Research, Singapore, Singapore; University of Patras, Greece

## Abstract

Universal phenotyping techniques that can discriminate among various states of biological systems have great potential. We applied 557 fluorescent library compounds to NCI's 60 human cancer cell-lines (NCI-60) to generate a systematic fluorescence phenotypic profiling data. By the kinetic fluorescence intensity analysis, we successfully discriminated the organ origin of all the 60 cell-lines.

## Introduction

A principal challenge for functional genomics is to identify genotypes that are associated with a specific phenotype. Recent advances in gene expression profiling and next-generation sequencing (NGS) technology have driven significant improvements in high-throughput genotyping. [1], [2] However, unlike the well established genotyping platforms, there is no standard quantitative methods for phenotyping yet. [3] Phenotypes can be defined at many different levels; e.g., biochemical, or physiological characteristics which can be measured at a cellular level, or behaviour or clinical history at an organism level. [4] Each phenotypic measurement has been used for further study on a case-by-case basis. For instance, behaviour and brain imaging pattern have frequently been used for phenomic character in neuroscience, [5]–[8] and CD (cluster of designation)-marker based biochemical phenotypes are extensively used to discriminate between cell types in various fields. [9], [10] However, there are not yet universal phenomic parameter that can be applied to diverse screening formats. [11]

We envisioned that a fluorescent probe library would have great potential to identify a set of universal phenomic parameters due to their uniform phenotypic readout (fluorescence signal), easy dose controls, high sensitivity to micro-environments, and structural diversity at the molecular level. Our group has recently developed fluorescent libraries and reported a series of imaging probes in glucagon secreting cells, [12] differentiated myotube cells, [13] and pluripotent stem cells. [14] Even in case the intracellular targets are not clearly defined, the phenotypic signature could be used for a variety of applications. [15] More importantly, depending on the properties of the fluorescent probe, high content information could be extracted from a single fluorescence image. [16], [17] Accordingly, fluorescent phenotype profiling using synthetic probes could reveal subtle differences in biochemical characteristics. In this paper, we report the first fluorescence intensity-based cellular phenomic profiling study using a diversity-oriented fluorescence library (DOFL) and NCI-60 cancer cell lines.

To demonstrate the phenomic scale of fluorescence image-based screening methodology, 60 human cancer cell lines (NCI-60) of the National Cancer Institute's Developmental Therapeutics Program were chosen. These cells were selected based on the following the two reasons. (i) it is the largest standard collection of broad cancer cell types. The NCI-60 set contains cancers of 9 different origins, including leukemia, melanomas, renal, breast, colon, lung, CNS, prostate, and ovarian carcinomas. (ii) rich biochemical background information about the collection is available to public. The characteristics of the NCI-60 set have been profiled at the genomic, [18], [19] proteomic, [20] and drug effect level, [21]–[23] and those data can be easily applied to further “omics” studies. While various profiling approaches have been widely explored to characterize the NCI-60 cell-lines, a systematic fluorescent probe-based profiling has not been pursued yet.

## Results and Discussion

### Fluorescent probes and fluorescence phenotyping

**Figure 1:**
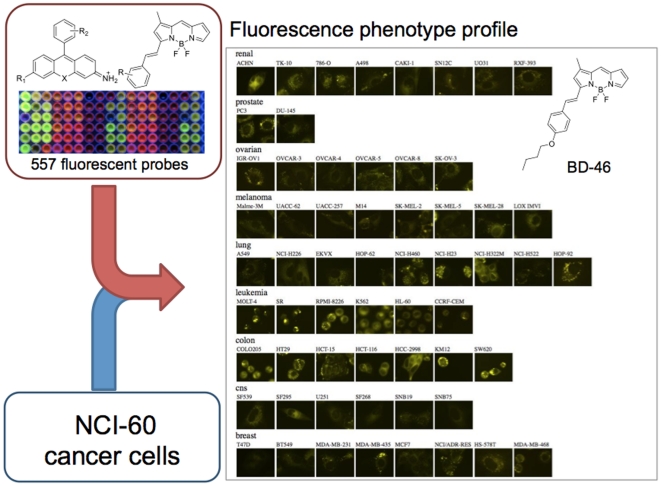
Fluorescence phenotype profiling workflow and representative fluorescence phenotype profile; BD-46 (500 nM) was added to 60 human cancer cell-lines and fluorescence phenotype profiles were measured after 1 h using automated fluorescence microscopy.

Combination of rosamine (**RS**) and BODIPY (**BD**) fluorophore libraries (240 rosamine [24] and 317 BODIPY [12] compounds, 557 compounds in total) were used to generate fluorescence phenotyping data. As previously reported, these compounds showed good cell permeability, and broad absorbance (**λ**_abs_ = 480∼616 nm) and fluorescence emission (**λ**_em_ = 530∼656 nm) ranges with a broad structural diversity. To maximize the information from the cell based assays, we utilized a whole cell fluorescence image-based screening platform, which would provide integrated cellular characteristics, so called high-contents screening. The large-scale profiling was performed using an automated fluorescence microscope system (ImageXpress Micro, Molecular Devices, Inc.). NCI-60 cells were plated in thin-bottom 384-well plates at 70% confluency, and 557 fluorescent compounds were added to different wells at two final concentrations of 250 nM and 500 nM. The fluorescence images were obtained both in the FITC and the TRITC channels to cover the broad range of spectra of the probes. Fluorescent images were taken from two independent sites of each well for 3 time points (1 hour, 24 hours and 48 hours), and all the experiments were duplicated. All together, 1,604,160 images (557 probes×60 cancer cells×2 concentrations×2 channels×2 sites×3 time points×2 duplicates) were collected. A representative fluorescence image data of **BD-46** is presented in Figure 1 and the details of the plate design is described in Figure S1. From these fluorescence images, many different kinds of features, such as morphology, texture, or intensity could be extracted and used as a phenotypic signature. [25] Notably, the staining intensities and the pattern of localizations varied in individual cell-lines, even though all cells were stained with an identical amount of **BD-46**.

### Quantitative fluorescence intensity profiling of NCI-60 cell lines

**Figure 2:**
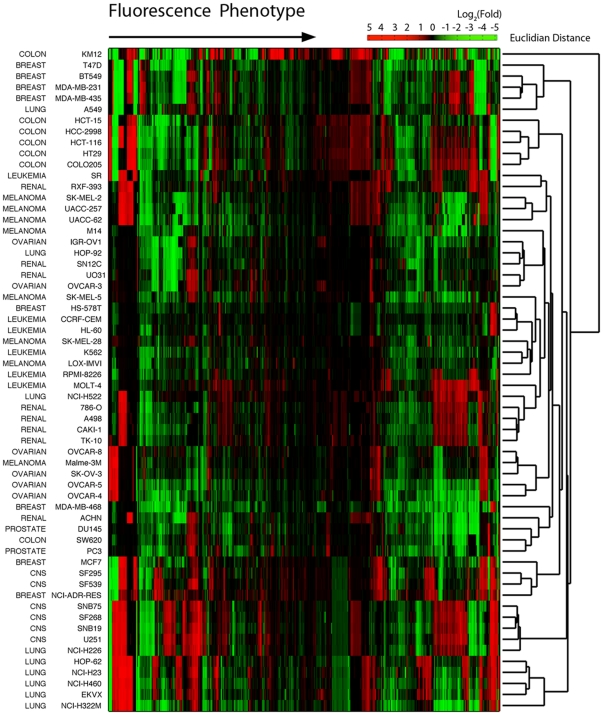
Hierarchical clustering of the fluorescence response phenotype. Fluorescence intensity fold changes were clustered for the 557 fluorescent probes (x-axis) across the 60 cancer cell lines (y-axis). Fold = (fluorescence intensity at 48 h incubation)/(fluorescence intensity at 1 h incubation).

Among various possible parameters, we focused on the fluorescence intensity of the cells, since the image processing method was straightforward and the processed data was less dependent to the analysis algorithm than others. To further suppress the artifact from the batch-to-batch variations (this is especially important in this study due to the long data acquisition time over 2 years), we decided to use fluorescence intensity fold change over the time (intensity kinetic profile), rather than the absolute value of the intensity. Firstly, we measured the mean values of the fluorescence intensity of the cells, and the intensity pattern for different cell-lines were analyzed by their intensity kinetic profile (fold change of the intensity values at 48 h over 1 h; the data processing protocols are described in the experimental methods). Then, we analyzed the overall fluorescence intensity pattern change of the NCI-60 set of cell-lines by heatmap analysis and hierarchical clustering (Fig. 2). In general, lung, colon, and ovarian cancer cells were well classified by the simple clustering. On the other hand, from a perspective of chemical structure, fluorescent compounds that shares same classes of xanthone derivative structure (e.g. **RS-A**, **RS-E**, and **RS-K** series) exhibited similar response pattern in the cell lines for same cancer origin (Fig. S2).

Interestingly, a remarkable signature was observed for the **RS-K** series of compounds. These compounds initially showed low fluorescence intensity, but after 24 h, their fluorescence was dramatically increased only in specific set of cell-lines (HCT-116, HT29, COLO205, HCC-2998, EKVX, HOP-62, NCI-H460, NCI-H322M, and NCI-H23). Moreover, fluorescence response profiles were highly correlated with the probe structure and cancer cell type (Fig. S2). For example, a series of **RS-K** compounds (**K1**, **K3**, **K4**, **K14**, **K15**, **K16**, and **K17**) exhibited fluorescence turn-on effects in lung and colon cancer cells (Fig. S3), and **RS-E** compounds (**E1**, **E3**, **E7**, and **E29**) showed turn-on responses in lung cancer cells (Fig. S4). Although the fluorescence response of **RS-K** and **RS-E** series increased for those specific cancer cells, each compounds showed subtle but distinct signature. In particular, **RS-K** series compounds showed turn-on effects towards broad ranges of lung cancer cell, but they differs response pattern depending on the 9-phenyl ring structure of rosamine (Fig. S3). It is also noteworthy to point out some of these probes distinguish specific cell lines from identical cancer origin. **RS-E1**, **RS-E3** and **RS-E7** compounds selectively turned on only in ACHN cells among renal cancer cells (Fig. S5). Moreover, they exhibited specific response to SW60 cells, but not for the rest of 6 colon cancer cells.

**Figure 3:**
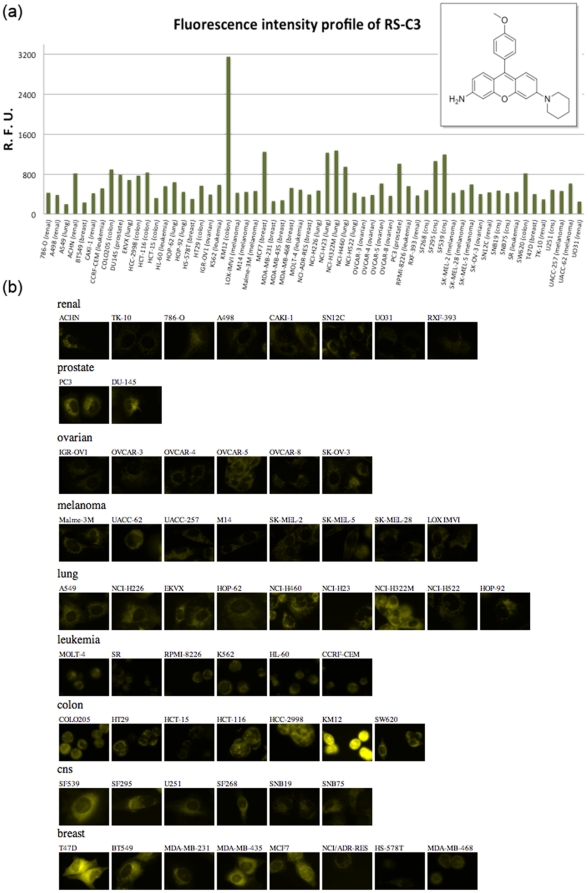
Fluorescence intensity profile of RS-C3 (500 nM) against NCI-60 cells after 24 h incubation and the corresponding fluorescent images. (a) bar graph of kinetic fold change (b) fluorescence images of 60 cancer cell lines.

Another cell line specific profile was observed with the **RS-C3** compound. **RS-C3** induced a strong fluorescence turn-on effect only in KM12 cells, a human colon cancer cell line, among 60 cell lines (Fig. 3). Compared with the average fluorescent intensities in other NCI-60 cells, **RS-C3** exhibited an exceptional 5.64-fold higher intensity in the KM12 cells. To explore the functional characteristic of the KM122 cells that induce selective fluorescence phenotype, we analyzed mRNA expression data of the NCI-60 cell lines. Based on the mRNA expression data of GSE5846, differentially expressed genes (DEGs) that up-regulated in KM12 cells (Log_2_(Fold) >1.5) were selected, and their gene annotation information were analyzed. KEGG pathway and gene ontology analyses identified five pathways that exhibit significant difference (*P* value <0.001) between KM12 cells and all the other NCI60 cells, such as cell communication, hematopoietic cell lineage, urea cycle and metabolism of amino groups and p53 signaling pathway (Table S3, S4). With these combinations of DEGs expression, it is possible to discriminate KM12 cells out of other NCI60 cell lines, and up-regulated profile of these genes are unique functional signature of KM12 cells. Since fluorescence phenotype of **RS-C3** shows collective information of such DEGs profile within a single fluorescence image as intensity changes, this probe has potential for sensing similar functional signature of other cell lines. While it was not clear at the moment what kinds of endogenous biomolecule are directly interacting with this probe, it could be utilized to visually identify a colon cancer cell line (KM12) among 60 cancer cell lines after 24 h pre-incubation.

### Discrimination of cancer cell origin

**Figure 4:**
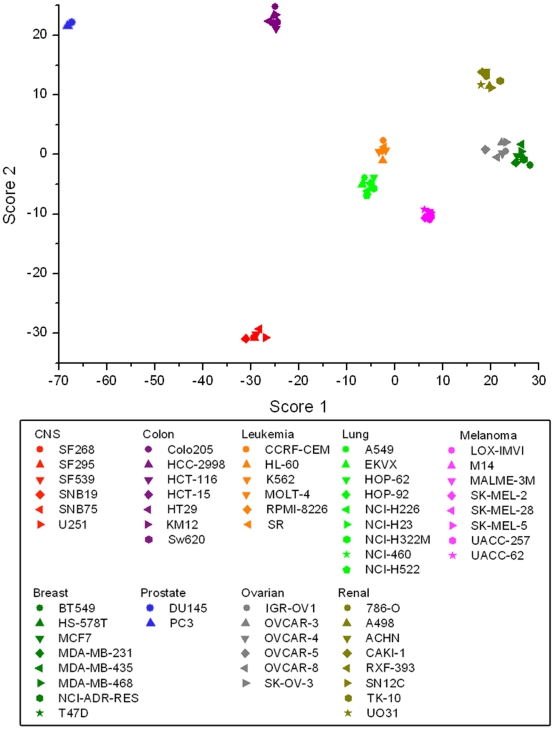
LDA score plot for the intensity kinetic profiles. x-, and y-axis represents the highest and 2^nd^ highest LDA scores by cell scoring function (Table S2). The 60 cancer cell lines were labelled according to the origin of cancer type; CNS: red, colon: purple, leukemia: orange, lung: green, melanoma: pink, breast: dark green, prostate: pale pink, ovarian: grey, and renal: olive.

For quantitative pattern analysis, the intensity kinetic profiles were further examined using linear discriminant analysis (LDA) to classify the cancer cell types. LDA is a commonly used classification method especially for high dimensional data for reduction of dimensionality. By applying LDA to kinetic profiles, it was possible to evaluate fold change responses of each chemical probe for optimum cancer cell identification, and generate score function to predict the identity of cells even each probe does not show specific response to single cell line. The minimum fluorescent probe set that could discriminate 9 cancer cell types was chosen by a forward step-wise variable selection algorithm, and 37 differentially responding fluorescent probes were selected (Fig. S6). Surprisingly, all 60 cancer cells were successfully clustered on an LDA score plot (Fig. 4), and 98% were correctly assigned to each of the original groups using *jackknifed* cross-validation (Table S1). LDA scores are arbitrary unit values representing maximum identity prediction, and our result showed the first and second highest LDA scores were enough to discriminate 60 cell lines in terms of cancer origin (Table S2). It is worth noting that classification using a combination of both **BD** and **RS** probes was much more successful as determined by cross-validation than any combination of either **RS** or **BD** probes alone (Table 1). This result implies that the chemical diversity of the fluorescent probes used to generate the intensity profile significantly affected the results of the cancer type classification. Another interesting aspect of selected 37 probes set is that none of these probes are selective to single cell or cancer types. They showed unique response patterns for multiple cell-lines, and the target cell was not always limited within identical cancer types. We believe such selective responses originated by specific biochemical signature or integrated intact environments of target cell types.

**Table 1:** LDA results for different fluorescnt probe sets.

	BD Library	RS Library	BD & RS Library
Selected probe number	37/317	22/240	37/557
% Correct classification in *Jackknifed* cross-validation	90%	80%	98%

Detailed *Jackknifed* classification matrix results are shown in Table S1.

### Conclusion

In short, we report the first fluorescence intensity-based phenotype profiling of 60 human cancer cell-lines (NCI-60) using synthetic fluorescent probes. Structural diversity of the fluorescent probes seems to be a critical factor for the generation of distinct phenotypes, and our results showed that 60 cancer cell-lines are all successfully discriminated in terms of 9 cancer cell origins. The most emphasis in cancer research has been focused on pathogenesis and metastasis, and metastasis research has been stagnated because of a lack of reliable tracking technique that could visualize a specific origin of cancer cells. Fluorescence compounds that probe specific origin of cancer cell could not only provide a new window for metastasis research, but also be utilized for cancer diagnosis and progression monitoring. Although this study has limitations in that we performed identification of cancer cells origin using only *in vitro* cancer cell lines, our results provided a clue that combinations of fluorescent probes could distinguish complex and subtle differences of cancer cells. Based on the response pattern, the better identification result was achieved when more fluorophore scaffolds were used, and the fluorescence intensity profiles were highly correlated with the chemical structure of the fluorescent probes and cancer types. These findings demonstrate the practical utility of the diversity-oriented fluorescence library approach in universal cell phenotyping.

## Methods

### Diversity-oriented fluorescence library preparation

The rosamine and BODIPY library compounds were prepared according to previously reported protocols, and the compound structures and characterization data has been reported. [12], [24], [26] All compounds were stored at −20°C in microtilter plates in solid form, and stock solutions were made by dissolving the compounds in DMSO prior to screening.

### NCI-60 cancer cell culture

The NCI-60 cell-lines were obtained from the National Cancer Institute. All NCI-60 cell-lines were cultured as described in the distributor's manual. Briefly, cells were cultured in a high-glucose Dulbecco's Modified Eagle's Medium (DMEM) supplemented with 10% FBS and 1% antibiotics.

### Quantitative fluorescence intensity analysis

Mean values of the fluorescence intensities were calculated using Matlab 7.9.0 (R2009b). In our dataset, 4 images represented each experimental condition (2 duplicate×2 images/well). We combined the 4 images using the *cat* function in Matlab to optimize IO processing. To minimize the background effect, only the fluorescence intensity values for area that were positive for Otsu's threshold were used to calculate the average value. The *graythresh* and *im2bw* functions in the Matlab image processing module were used to calculate Otsu's threshold and the cell area selection. All quantitative image processing batch jobs were computed using a 16-node PC cluster for 3 days.

### Discriminant analysis

Linear discriminant analysis (LDA) was performed using fold change values of the fluorescence intensities between 48 hour and 1 hour incubation points. Probe selection was processed by forward-stepwise variable selection algorithum in SYSTAT (version 13).

### NCI-60 gene expression and pathway analysis

The mRNA expression patterns for the NCI-60 set of cell lines were downloaded from the NCBI gene expression omnibus (GEO) database. The GSE5846 data were analyzed using GenePlex v3.0 DEG finding and Pathway analysis modules (ISTECH, Inc.).

## Supporting Information

Figure S1 **Schematics of NCI-60 assay format.** We used 384 well plate for high throughput fluorescent imaging. 60 cells were divided into 13 subsets (each set contains less than 5 cell lines), and 2 fluorescent probes are tested with each of the subsets in an individual well plate. Since the culture media evaporate fast in edge wells, first and last two columns were not used for the assay.
(TIF)

Figure S2 **SAR of fluorescent probes in phenotype profile.** Hierarchical cluster of fluorescent response phenotype reveals structural relationship of fluorescent probe. Fluorescent intensity changes pattern of 557 fluorescent probes (x-axis) against 60 cancer cells (y-axis) were clustered. Fold = (fluorescent intensity after 48 h incubation)/(fluorescent intensity after 1 h incubation).
(TIF)

Figure S3 **Fluorescence intensity profile of RS-K series.** (a) Fluorescence response profile of **RS-K15** in NCI60 cells and fluorescence images of lung cancer cell lines. First and second row images were taken after 1 h and 24 h after probe treatment respectively. All 4 images for each experimental condition are shown. (b) Fluorescence intensity bar graph pattern of **RS-K** series towards 60 cancer cell line.
(TIF)

Figure S4 **Fluorescence intensity profile of RS-E series.**
(TIF)

Figure S5 **Cell line specific response of RS-E series compounds among renal and colon cancer origin.** Fluorescence intensity bar graph of **RS-E1**, **RS-E3**, and **RS-E7** compounds for cancer cells from (a) colon and (b) renal origin.
(TIF)

Figure S6 **Structures of 37 selected fluorescence probes from LDA.** Probes were choosed based on step-wise forward automatic variable selection algorithm with alpha-to-enter: 0.150 and alpha-to-remove: 0.150 criteria using SYSTAT v13.
(TIF)

Table S1 ***Jackknifed cross-validation*** **matrix for three set of fluorescent probes.**
(XLS)

Table S2 **LDA score function and coefficient of selected fluorescent probes.**
(XLS)

Table S3 **Selected KEGG pathways based on DEGs of KM12.** *P*-values were calculated by *Fisher's exact test*.
(XLS)

Table S4 Top 20 biological processes from gene ontology annotation of DEGs in KM12 cell.
(XLS)
