# Supplementary figures and images for: Identification of Cancer Cell-Line Origins Using Fluorescence Image-Based Phenomic Screening
Source: PLoS One. 2012 Feb 23;7(2):e32096. doi: 10.1371/journal.pone.0032096 (PMC3285665; doi:10.1371/journal.pone.0032096)

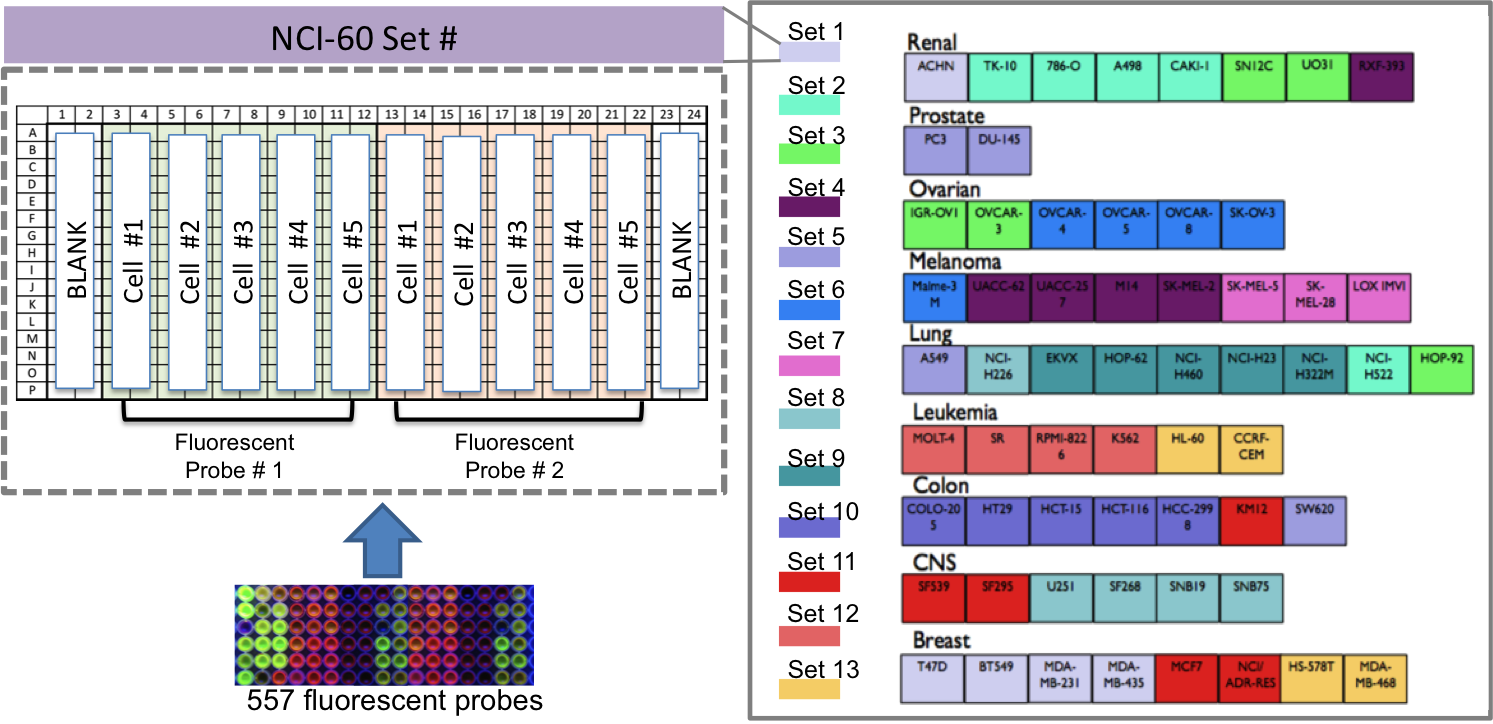

Supplement: Figure S1 — Schematics of NCI-60 assay format. We used 384 well plate for high throughput fluorescent imaging. 60 cells were divided into 13 subsets (each set contains less than 5 cell lines), and 2 fluorescent probes are tested with each of the subsets in an individual well plate. Since the culture media evaporate fast in edge wells, first and last two columns were not used for the assay. (TIF) [file pone.0032096.s001.tif]

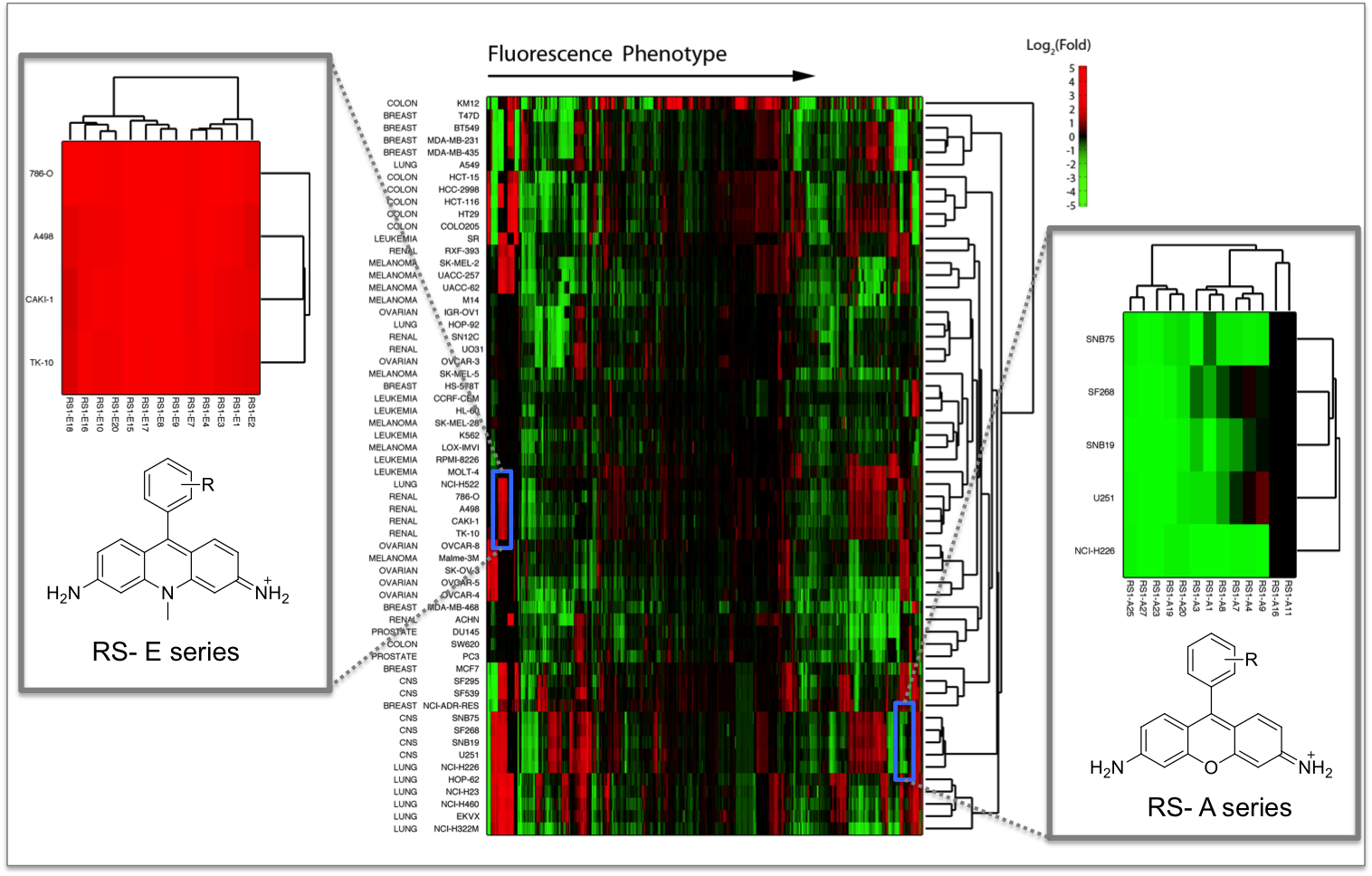

Supplement: Figure S2 — SAR of fluorescent probes in phenotype profile. Hierarchical cluster of fluorescent response phenotype reveals structural relationship of fluorescent probe. Fluorescent intensity changes pattern of 557 fluorescent probes (x-axis) against 60 cancer cells (y-axis) were clustered. Fold = (fluorescent intensity after 48 h incubation)/(fluorescent intensity after 1 h incubation). (TIF) [file pone.0032096.s002.tif]

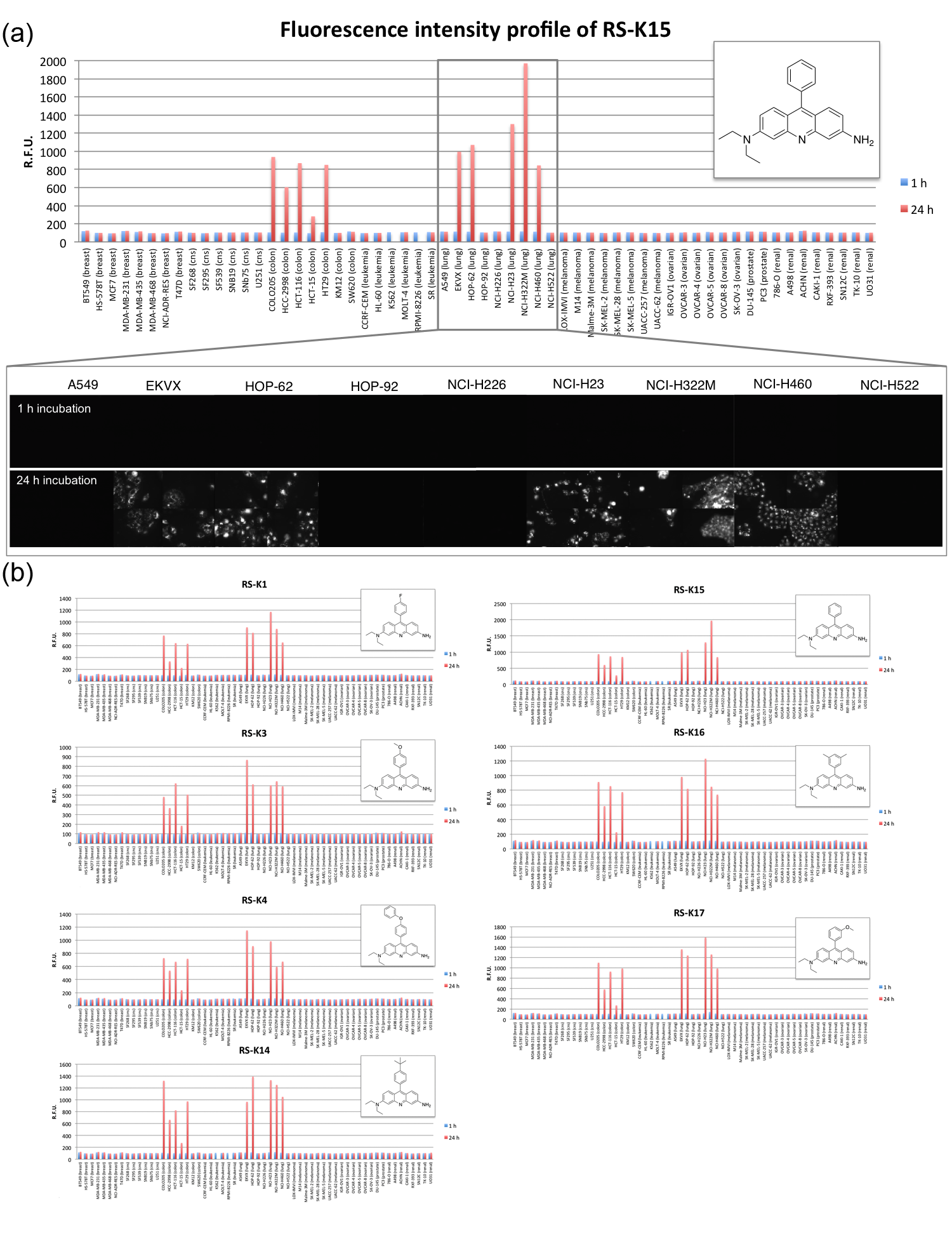

Supplement: Figure S3 — Fluorescence intensity profile of RS-K series. (a) Fluorescence response profile of RS-K15 in NCI60 cells and fluorescence images of lung cancer cell lines. First and second row images were taken after 1 h and 24 h after probe treatment respectively. All 4 images for each experimental condition are shown. (b) Fluorescence intensity bar graph pattern of RS-K series towards 60 cancer cell line. (TIF) [file pone.0032096.s003.tif]

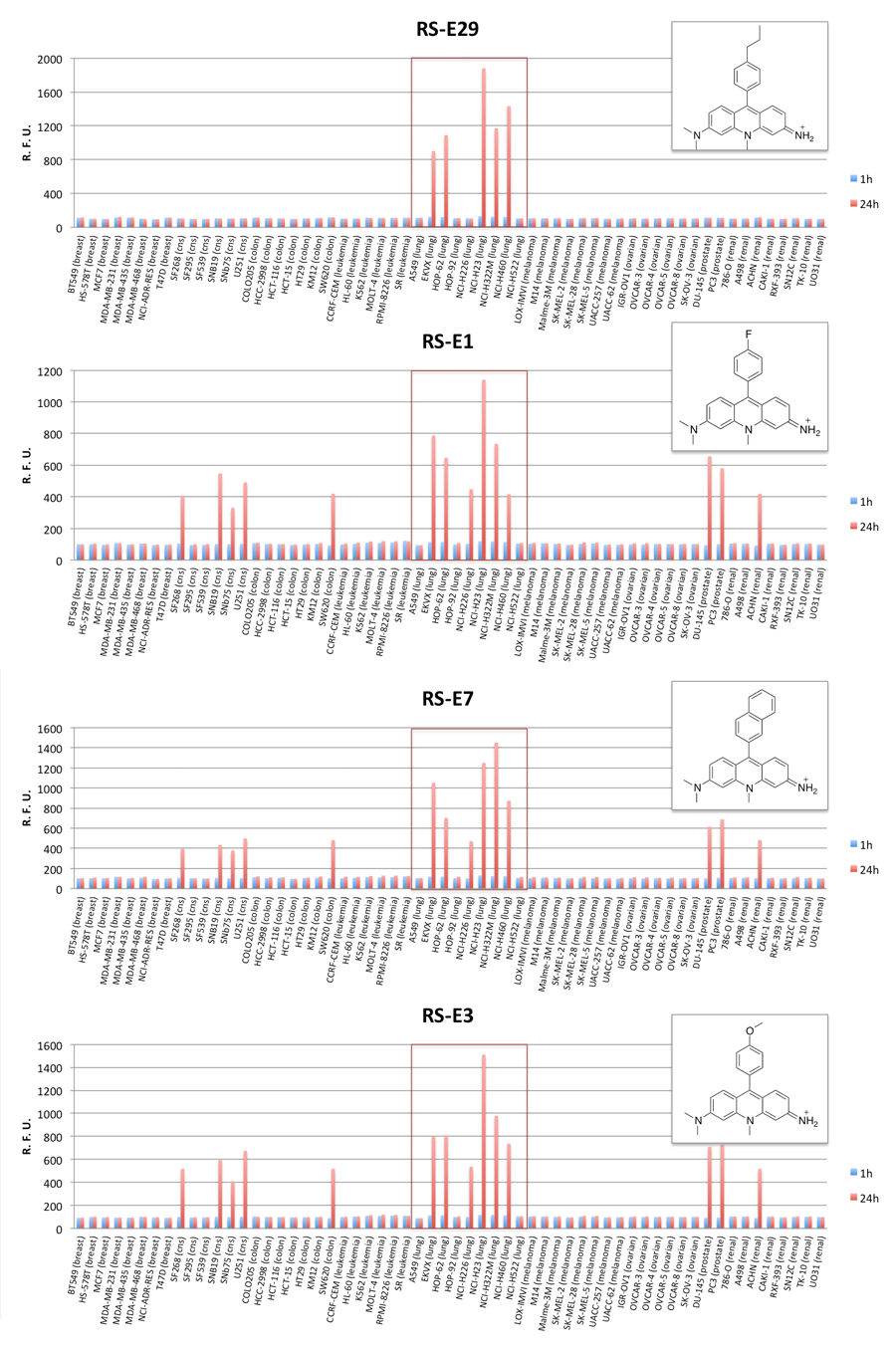

Supplement: Figure S4 — Fluorescence intensity profile of RS-E series. (TIF) [file pone.0032096.s004.tif]

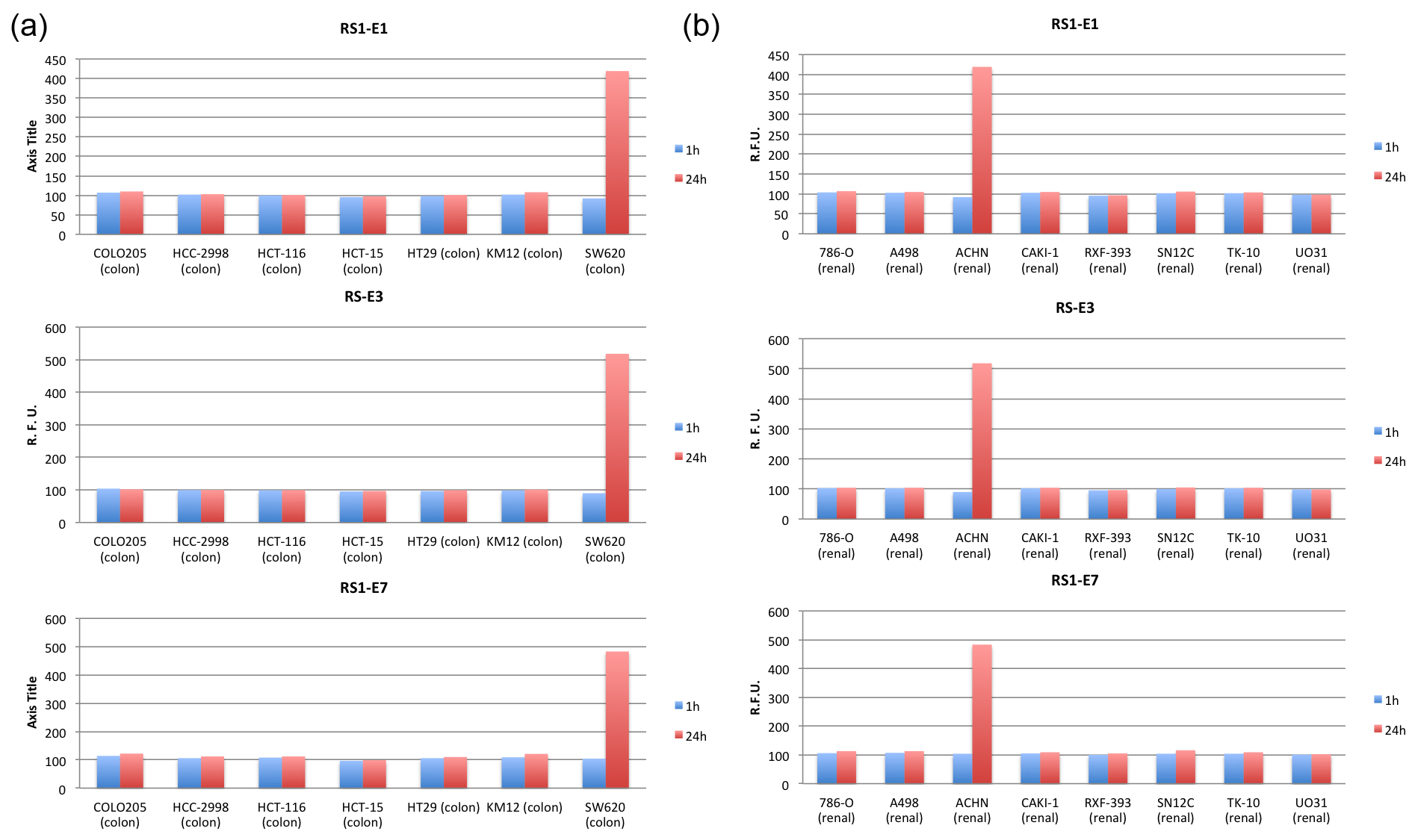

Supplement: Figure S5 — Cell line specific response of RS-E series compounds among renal and colon cancer origin. Fluorescence intensity bar graph of RS-E1, RS-E3, and RS-E7 compounds for cancer cells from (a) colon and (b) renal origin. (TIF) [file pone.0032096.s005.tif]

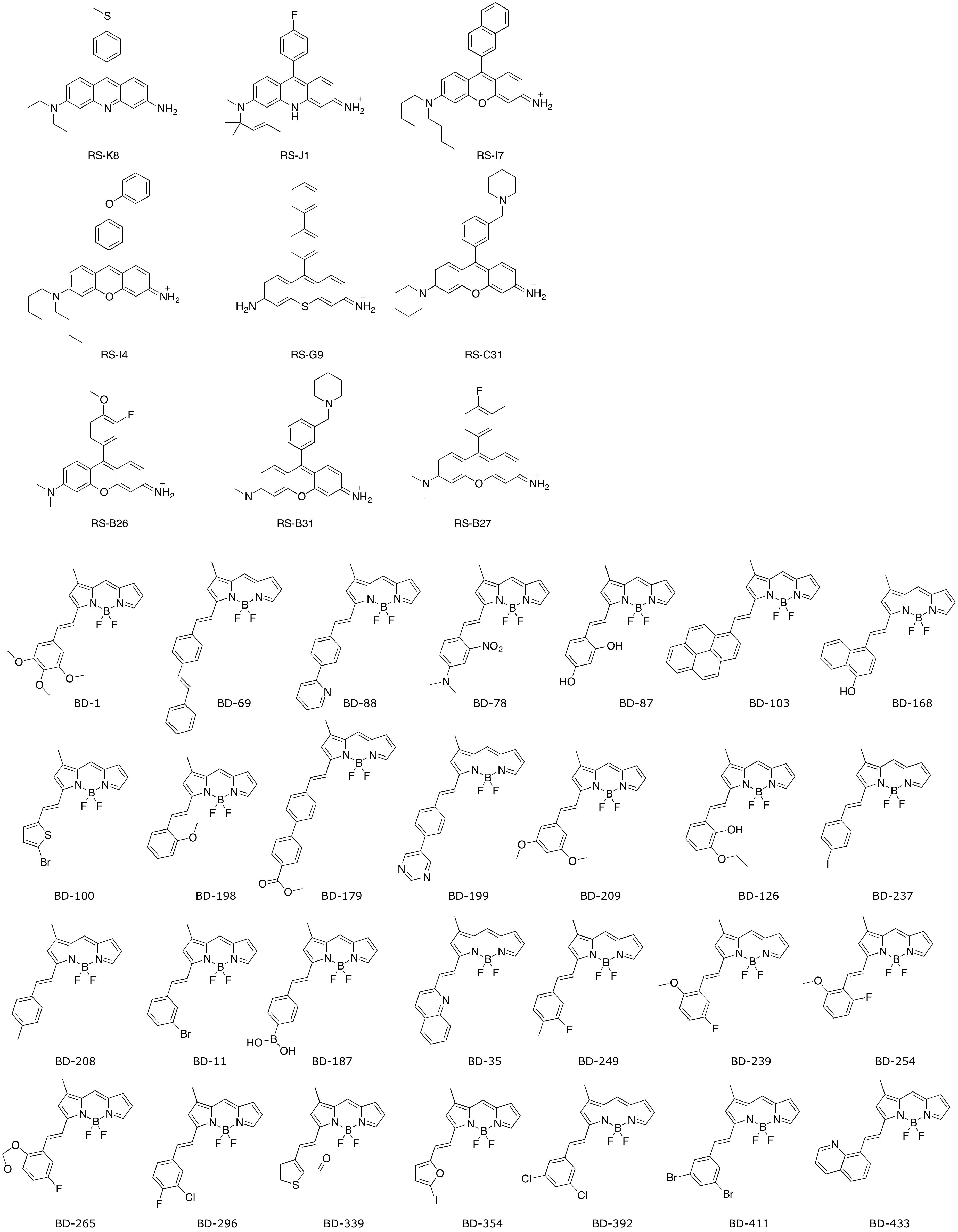

Supplement: Figure S6 — Structures of 37 selected fluorescence probes from LDA. Probes were choosed based on step-wise forward automatic variable selection algorithm with alpha-to-enter: 0.150 and alpha-to-remove: 0.150 criteria using SYSTAT v13. (TIF) [file pone.0032096.s006.tif]
